# Mitogen-activated protein kinases (MAPKs) are modulated during in vitro and in vivo infection with the intracellular bacterium *Burkholderia pseudomallei*

**DOI:** 10.1007/s10096-017-3038-0

**Published:** 2017-08-30

**Authors:** R. V. D’Elia, R. J. Saint, S. L. Newstead, G. C. Clark, H. S. Atkins

**Affiliations:** 1 CBR Division, Defence Science and Technology Laboratory, Porton Down, Salisbury, SP4 0JQ UK; 2 University of Exeter, Exeter, UK

## Abstract

*Burkholderia pseudomallei* is a Gram-negative intracellular bacterium that causes the disease melioidosis. The disease can be fatal if left untreated or when antibiotic therapy is delayed and total clearance of the pathogen from the host is often not accomplished with current therapies. Thus, new therapeutic approaches for the treatment of infections caused by *B. pseudomallei* are required. To better understand host responses to *B. pseudomallei* infection, the activation of key proteins involved in the TLR inflammatory cascade was measured by western blotting. Activation of the mitogen-activated protein kinases (MAPKs) p38 and ERK were both significantly altered during both in vitro and in vivo infection. In considering an approach for therapy of *B. pseudomallei* infection the inhibition of ERK was achieved in vitro using the inhibitor PD0325901, along with decreased TNF-α production. However, the reduction in phosphorylated ERK and TNF-α release did not correspond with decreased bacterial replication or enhance clearance from infected macrophages. Despite this apparent lack of effect on the intracellular growth of *B. pseudomallei* in vitro, it is not clear what effect inhibition of ERK activation might have on outcome of disease in vivo. It may be that decreasing the levels of TNF-α in vivo could aid in reducing the overactive immune response that is known to ensue following *B. pseudomallei* infection, thereby increasing host survival.

## Introduction

The innate immune response is one of the first lines of defence from infection triggered by a range of host-pathogen interactions. The first step is represented by the binding of pathogen associated molecular patterns (PAMPs; e.g. lipids, proteins, nucleic acids) to pattern recognition receptors (PRRs) such as Retinoic acid (RIG)-like receptors, Nod-like receptors (NLRs) and Toll-like receptors (TLRs) which subsequently induce signalling cascades generating immune responses [1–3].

Numerous human TLRs have been identified and shown to be expressed on various cell types including dendritic cells, neutrophils, alveolar macrophages and epithelial cells [4–7]. TLRs detect specific sets of PAMPs (e.g. TLR2 detect lipoprotein, TLR4 detect lipopolysaccharide; LPS). These TLRs play an important role in the development of numerous infectious diseases including melioidosis caused by the Gram-negative bacterium *Burkholderia pseudomallei*. Specifically, the TLR2 and TLR4 pathway (including the downstream inflammatory cascade and soluble factor proinflammatory responses) has been demonstrated to be important in disease formation both within in vitro cellular, knock-out and wild type experimental models of infection and within human cases of melioidosis [8–14].

Production of pro-inflammatory cytokine and chemokines requires both the activation and binding of TLRs along with the subsequent activation of multiple protein-protein complexes via two major signalling pathways: MyD88 and TRIF [15]. The activation/phosphorylation of mitogen-activated protein kinases (MAPKs) downstream of MyD88 and TRIF converts extracellular stimulations to intracellular responses via cascade proteins such as p38, ERK and JNK required in order to mount a host response during bacterial and viral infections [16–19]. The expression of p38 and ERK in Raw 264.7 macrophages infected *B. pseudomallei* strain E8 has previously been reported [20] and, in addition, the inhibition of p38 was found to reduce the invasion of human alveolar lung epithelial cells by *B. pseudomallei* strain 844 [21]. Here we sought to build upon these reports by determining the expression and activation of the key MAPKs p38 and ERK in vitro and, uniquely, to validate these responses in vivo within a mouse model of infection for *B. pseudomallei* strain K96543. The work aimed to determine whether manipulation of MAPKs offers potential as a novel therapeutic strategy for treating melioidosis.

## Materials and methods

### Bacterial culture

*B. pseudomallei* strain K96243 was cultured from frozen stocks in L-broth at 37 °C overnight with shaking. Subsequently, the suspension was adjusted using phosphate-buffered saline (PBS) to an optical density at 600 nm of 0.35, with an estimated bacterial density of approximately 1 × 10^8^ colony forming units (CFU) per ml. Bacteria were enumerated on agar plates following serial dilution (1:10) of samples.

### In vivo model of infection with *B. pseudomallei*

Six-to-eight week old female BALB/c mice (Charles River, UK) were transferred to a class III rigid isolator and given unlimited access to food and water. Mice were challenged with 460 CFU (calculated retained dose) of *B. pseudomallei* strain K96243 by the aerosol route using the Henderson apparatus [22] and a Collison nebuliser. Mice were checked twice daily and scored for clinical symptoms to identify suitable humane end points. Groups of five mice were culled at 0, 3, 10, 24 and 36 h post-infection and the lungs were removed and homogenised through a 40-μm cell sieve before filter sterilisation through a 0.2-μm syringe filter. The activation of signalling factors and the pro-inflammatory cytokine response was assessed in the homogenised and sterilised lung samples at each time point. All animal studies were carried out in accordance with the UK Scientific Procedures Act (1986).

### In vitro macrophage model of infection

The murine alveolar macrophage-like MH-S cell line was obtained from the European Collection of Cell Cultures (ECACC). Cells were maintained in endotoxin-free Roswell Park Memorial Institute medium (RPMI) (Gibco) supplemented with 10% FCS (Gibco) and 2 mM L-glutamine (Sigma). MH-S cells were seeded into 6-well plates at approximately 1 × 10^6^ cells/ml and incubated overnight at 37 °C. The supernatants were removed and replaced with 1.5 ml of *B. pseudomallei* at an MOI of 10. Plates were incubated at 37 °C for 30 min to allow for internalisation of the bacteria and then the supernatants were removed and replaced with 1.5 ml of fresh media. The plates were incubated at 37 °C and, at a range of time points post-infection, supernatants were removed and stored at –20 °C for cytokine analysis. The cells were washed once with chilled PBS and then lysed by adding 350 μl PhosphoSafe™ extraction reagent (Merck) and incubating on ice for 5 min. The cellular lysates were used for either bacterial enumeration on agar plates or centrifuged at 13,000 rpm for 5 min to remove cell debris before being stored at –20 °C for subsequent analysis by western blotting.

### ERK inhibition assay

MH-S cells were infected with *B. pseudomallei* at an MOI of 10 (equivalent to *t* = −30 min) and the cells were allowed to internalise bacteria for a further 30 min. Control cells were left uninfected (naïve). The media was removed, cells were washed with PBS and fresh media applied (equivalent to *t* = 0 h). The ERK inhibitor PD0325901 (InvivoGen @ 0.05 μM) was added to the cells at either 0 h or 2 h after the cells were washed of non-internalised bacteria. At multiple time points after infection, supernatants and cell lysates were collected for cytokine analysis, screening for signalling protein activation and bacterial enumeration.

### Detection of intracellular proteins via western blotting

Proteins were transferred from polyacrylamide gels onto Invitrolon™ Polyvinylidene fluoride (PVDF) membranes (Invitrogen, UK) using a Novex® Semi-Dry Blotter (Invitrogen, UK) according to manufacturer’s instructions. Methods followed previously published work [23] with the following specific primary antibodies (p38: p38 p38α MAPKinase #9218, P-p38: Phospho-p38 MAPKinase #9211, ERK-1: p44 MAPKinase #4372, ERK-2: p42 MAPKinase #9108 and P-ERK-1/2 Thr202/Tyr204: Phospho-p44/42 MAPK #9101, New England Biolabs, UK). IRAK-1 (Abcam, ab238), TAK-1 #4505, SAPK/JNK #9252, IKK-α #2682, IKK-β #2684, IKK-γ #2685, IκBα #9242 and IκBβ #9248 (New England Biolabs, UK) were also tested but data is not shown. The films were computationally analysed by densitometry which allocates a value to each band relating to its size and optical density, using a GS-800 Imaging Densitometer (Bio-Rad, US) combined with Quantity One® analysis software v 4.2.1 (Bio-Rad, US). Data was normalised to the β-actin (#4967, New England Biolabs) value from the same sample.

### Measurement of soluble inflammatory markers

Levels of IFN-γ, IL-6, IL-10, IL-12p70, TNF and MCP-1 in both in vitro and in vivo infection samples were measured via Cytometric Bead Array (Becton Dickinson™) in accordance with manufacturer’s instructions, with the additional initial step of fixing the lung samples in 4% paraformaldehyde in PBS for at least 24 h at 4 °C. Briefly, following fixing, samples were incubated with the combined capture bead cocktail, and PE detection antibodies for 2 h. Samples were washed and re-suspended in FACS buffer. Cytokine concentrations were measured via quantification of PE fluorescence of samples in reference to a standard curve using a BD FACS CANTO flow cytometer (Becton Dickinson™).

### Statistical analysis

All transformations of data and statistical tests were performed using GraphPad Prism® 5.01 software. Data from cytometric bead arrays were analysed using PRISM, by fitting a quadratic regression to the standard curves and reading the samples as unknowns.

## Results

### In vitro response to *B. pseudomallei* infection

**Fig. 1:**
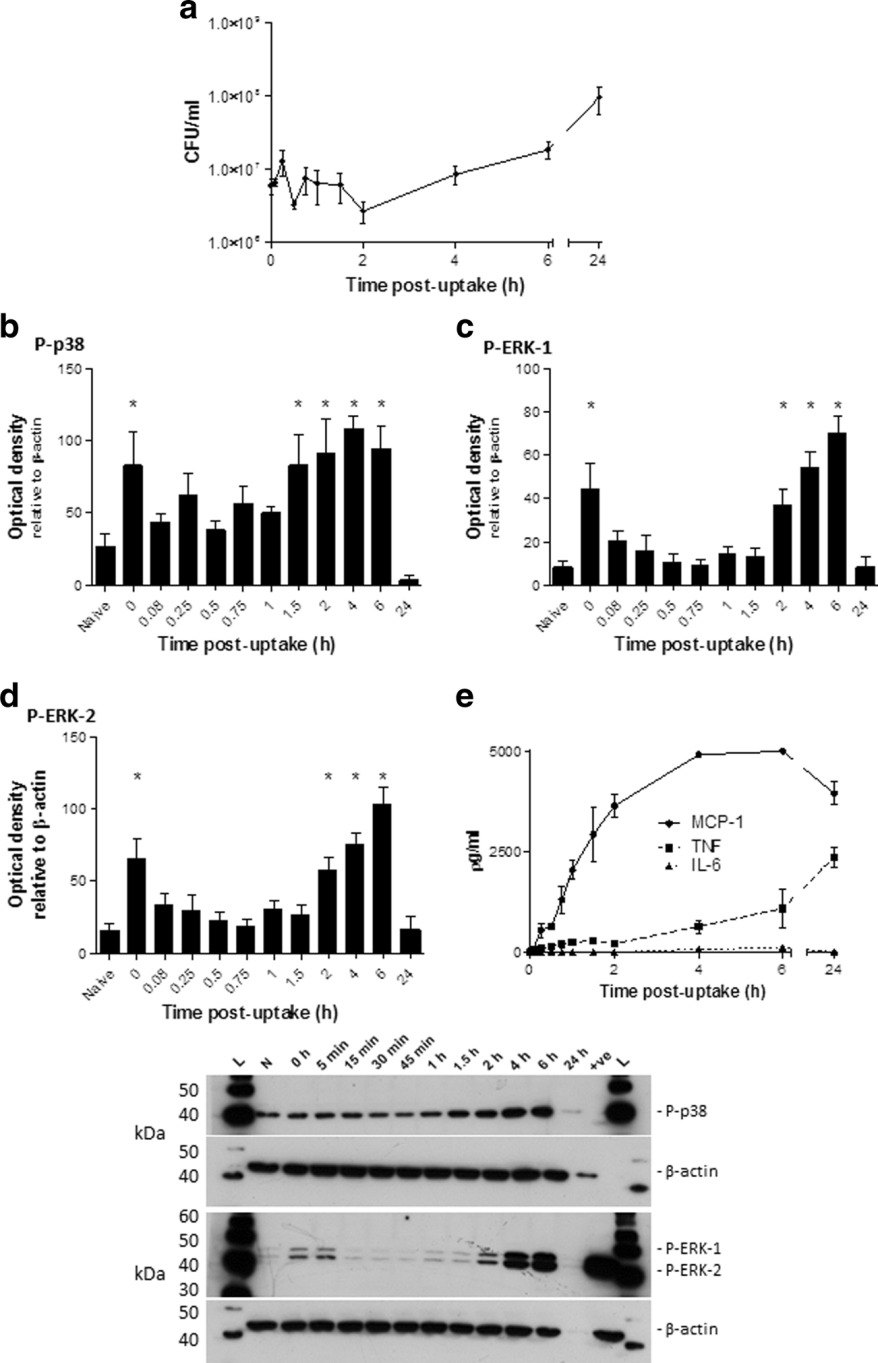
Characterisation of the in vitro response to *B. pseudomallei.* MH-S cells were infected with *B. pseudomallei* K96423 at an MOI of 10 and incubated for 30 min at 37 °C to allow internalisation of the bacteria. At various time points after infection bacteria were enumerated on LB plates (**a**) and levels of P-p38 (**b**), P-ERK-1 (**c**), and P-ERK-2 (**d**) were determined in cell lysates by western blotting. Levels of TNF, MCP-1 and IL-6 were measured in cell supernatants via CBA (**e**). In all cases, data shown is the combination of three independent experiments. Significant differences from naïve samples were determined using a one-tailed, unpaired t-test (* = *p* < 0.05). Error bars show 95% confidence intervals. Western blot image: L – MagicMark™ ladder; N – naïve; +ve – positive control

As the lung is the primary organ associated with an inhalational *B. pseudomallei* infection, with as few as ten CFU causing disease via this route [24], a lung-derived (MH-S) cell line was used to determine the in vitro response to infection with *B. pseudomallei* strain K96423. We previously showed MH-S cells to produce a similar response to ex vivo lung cells and thus they were considered an appropriate initial model [23, 25]. Cells were infected with *B. pseudomallei* and at multiple time points post-infection the bacteria were enumerated, signalling protein activation was measured in cell lysates and the pro-inflammatory response was measured in cell supernatants (Fig. 1). Bacterial numbers decreased in the first 2 h following infection and subsequently increased through to 24 h (Fig. 1a). *B. pseudomallei* infection induced a rapid but transient increase in P-p38 at 0 h (30 min after infection) which was suppressed at 0.08 h (5 min) (Fig. 1b). A second increase in P-p38 occurred at 1.5 h and this activation was sustained to the 6 h time point. Another MAPK protein, ERK-1/2, showed a similar bi-phasic activation response comprising an early transient activation followed by a late sustained activation (Fig. 1c and d). Of the six cytokines screened for only three (MCP-1, TNF and IL-6) were found to be produced during infection (Fig. 1e); IFN-γ, IL-10 and IL-12p70 were not detected. MCP-1 was rapidly secreted during the early stages of infection and reached maximal levels of detection (5000 ρg/ml in the assay used) by 4 h. TNF was secreted at a slower rate than MCP-1 but, by 24 h post-infection, was detected at ∼2500 ρg/ml. In comparison, IL-6 was not readily secreted and was not elevated above baseline levels at 4 h and 6 h time points.

### In vivo response to *B. pseudomallei* infection

**Fig. 2:**
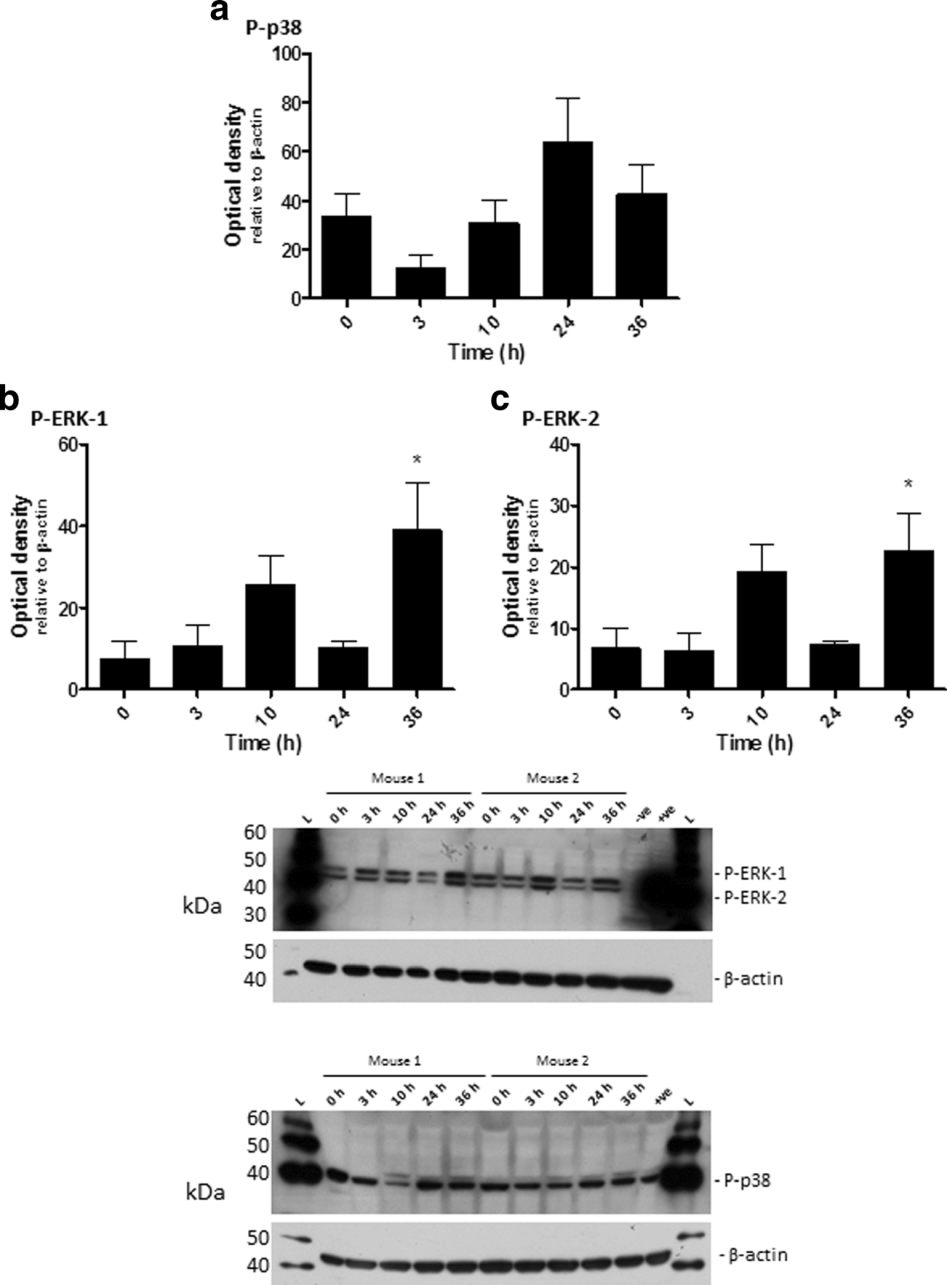
Characterisation of p38 and ERK in vivo response to *B. pseudomallei*. Groups of five mice were challenged with 460 CFU of *B. pseudomallei* K96423 via the intranasal route and five mice were culled at each time point. Levels of P-p38 (**a**), P-ERK-1 (**b**) and P-ERK-2 (**c**) in the lungs of infected mice were determined at 0, 3, 10, 24 and 36 h after infection. Significant differences from naïve samples were determined using a one-tailed, unpaired t-test (* = *p* < 0.05). Error bars show 95% confidence intervals. Western blot image: L – MagicMark™ ladder; N – naïve; +ve – positive control

To determine whether the bi-phasic activation of ERK and p38 observed in vitro within MH-S cells was mirrored in vivo, Balb/c mice were infected with *B. pseudomallei* K96423 via the intranasal route. At set time points mice were culled and the activation of signalling proteins within lung homogenates was determined by western blotting. In contrast to the in vitro infection of MH-S cells, *B. pseudomallei* infection of Balb/c mice caused no detectable increase in P-p38 activation (Fig. 2a), although a trend towards activation was noted. Corresponding with the in vitro results *B. pseudomallei* infection caused a transient increase in P-ERK activation at 10 h post-infection followed by a second peak at 36 h post-infection (Fig. 2b and c). Consistent with the in vitro data, the TLR signalling cascade proteins IRAK-1, TAK-1, SAPK/JNK, IKK-α, IKK-β, IKK-γ, IκBα and IκBβ were not found to be significantly activated compared to the control group (data not shown).

### Effects of the inhibition of ERK activation by PD0325901 during infection

**Fig. 3:**
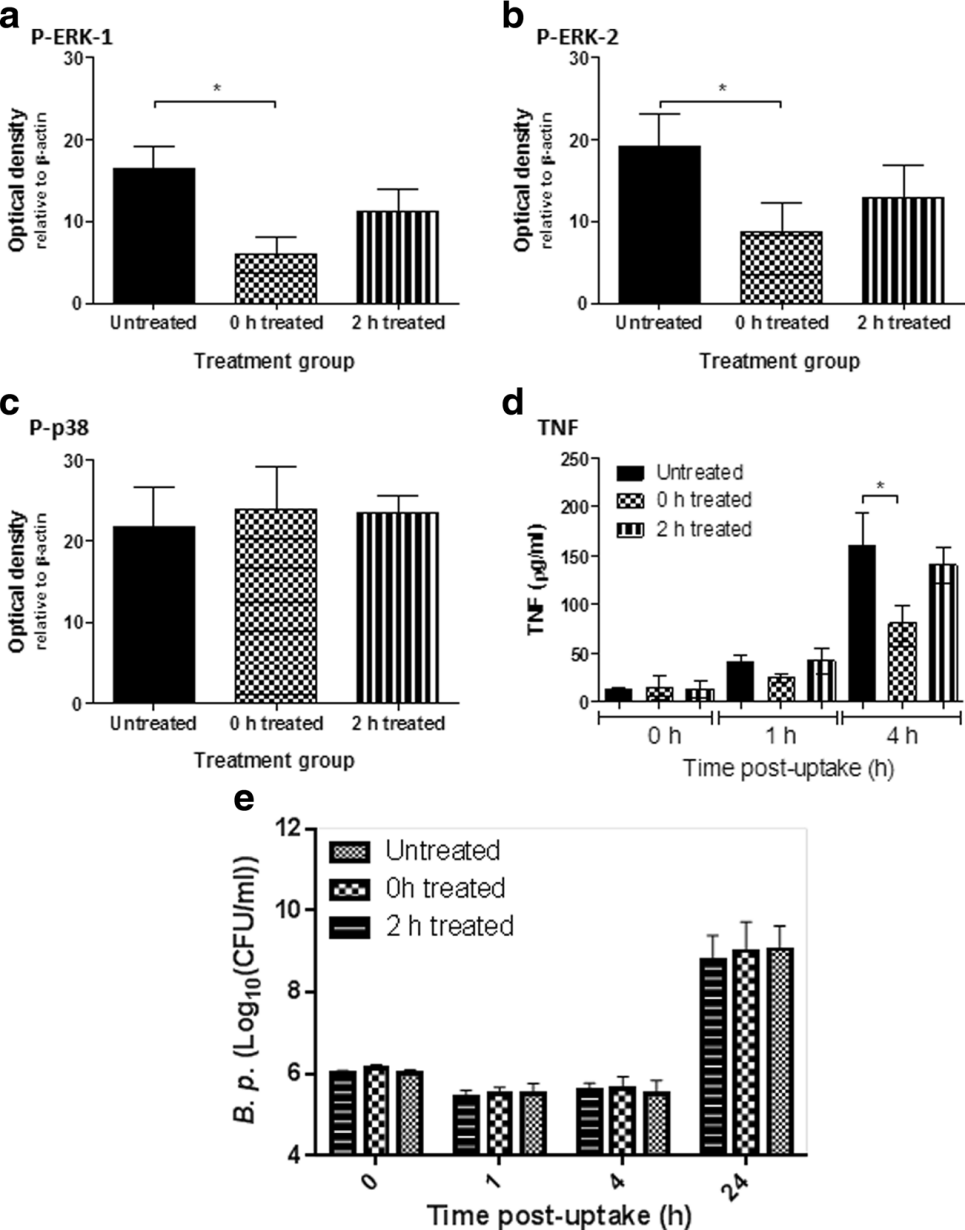
Treatment of infection with the ERK inhibitor, PD0325901. MH-S cells were infected with *B. pseudomallei* K96423 at an MOI of 10 and incubated for 30 min at 37 °C to allow uptake of the bacteria. The cells were treated with PBS (untreated), treated with PD0325901 at 0 h or treated with PD0325901 at 2 h after infection. Levels of P- ERK-1 (**a**), P-ERK-2 (**b**) and P-p38 (**c**) were determined in cell lysates collected at 4 h after infection by western blotting. Levels of TNF were measured in the cell supernatant via CBA at three time points after infection (**d**). Bacteria were enumerated on LB plates (**e**). In all cases, data shown is the combination of three independent experiments. Significant differences from naïve samples were determined using a one-tailed, unpaired t-test (* = *p* < 0.05). Error bars show 95% confidence intervals

Previous studies have observed that sustained ERK activation can lead to host cell apoptosis [26] and is used as a mechanism of viral propagation for the herpes simplex virus type 2 (HSV-2) [17]. A conceivable therapeutic strategy for treating a highly virulent intracellular bacterial pathogen such as *B. pseudomallei* might therefore be to inhibit ERK activation in order to re-balance the immune response and aid the resolution of infection. Several inhibitors of MAPKs have previously been assessed in vitro and in vivo with PD0325901 demonstrated to be a highly specific, potent ERK1/2 inhibitor [27]. In order to determine the effect of in vitro ERK inhibition, infected MH-S cells were treated with the PD0325901 inhibitor at 0 h (30 min after initial *B. pseudomallei* infection) or at 2 h (post-infection). When the cells were treated immediately after infection (i.e. at 0 h) there was a significant reduction in the phosphorylated levels of ERK at 4 h post-infection when compared to untreated controls (*p* < 0.05). In comparison, when PD0325901 treatment was delayed until 2 h post-infection the presence of phosphorylated ERK at 4 h was not significantly different to untreated control cells (Fig. 3a and b). To provide some assurance that PD0325901 was inhibiting the activation of ERK proteins, as opposed to other structurally homologous MAPKs, the activation of p38 was also measured. No significant inhibition of p38 activation was observed in cells treated with PD0325901 when compared to untreated controls (Fig. 3c). Since ERK activation was inhibited in this assay system, the indirect effects of ERK inhibition could be determined by measuring inflammatory cytokine secretion. Cells with PD0325901 administered at 0 h had significantly decreased TNF secretion after 4 h (*p* < 0.05) when compared to untreated controls (Fig. 3d). In contrast, the administration of PD0325901 at 2 h had no effect on TNF secretion. The presence of other inflammatory cytokines including IL-6, MCP-1, IFN-y, IL-10 and IL-12 were not significantly altered following treatment at either time point (data not shown).

Bacteria were enumerated within the MH-S cells in order to determine whether the effects of reducing ERK activation via PD0325901 administration led to differences in the intracellular growth and survival of *B. pseudomallei*. In this study no significant difference in intracellular bacterial numbers was detected within MH-S cells despite PD0325901 treatment (Fig. 3e).

## Discussion

The identification of the specific signalling proteins involved in the response to an infection can provide important information concerning the pathogenesis, mechanisms of survival and immune evasion used by pathogens [28]. This study has demonstrated that intracellular *B. pseudomallei* triggered temporal changes in the phosphorylation states of two MAPKs, p38 and ERK, and that these same patterns were also identified in vivo following the pulmonary infection of Balb/c mice with organism. In particular, we have shown significant increases in P-ERK in the latter stages of infection.

By investigating the temporal dynamics of signalling protein activation during infection of MH-S cells an initial increase in both P-p38 and P-ERK1/2 was evident following a 30 min incubation with the bacteria (*t* = 0 h). These proteins were quickly dephosphorylated returning to naive levels of activation until 2 h post-infection, whereupon a rapid and sustained activation of these kinases occurred. A recently published study also highlighted the potential importance of the phosphorylation of p38 and ERK following *B. pseudomallei* infection [20]. However, the previous study used a soil isolated strain E8 of *B. pseudomallei* and RAW 264.7 cells, whilst here we have used a clinical isolate in strain K96243 and an alveolar macrophage cell line (i.e. more relevant to pulmonary melioidosis). Crucially, our study has measured the phosphorylation state of p38 and ERK over a longer time course of infection with samples taken more frequently, allowing a greater characterisation of the biphasic nature of the phosphorylation state of p38 and ERK which may be essential in determining an optimum time to initiate a therapeutic intervention.

The temporal dynamics of ERK activation, in particular, may provide important information with respect to the pathogenesis of *B. pseudomallei*. Transient activation of ERK corresponds with host cell survival strategies whereas sustained activation is associated with cell death mechanisms [29, 30]. The transient activation of p38 and ERK demonstrated here indicates that during the early stages of infection with *B. pseudomallei*, host cells may adopt cell survival strategies. During latter stages of infection, the host cells may be in the process of cell death, as indicated by sustained ERK activation. This pattern of signalling protein activation points towards *B. pseudomallei* regulating ERK in order to delay the induction of cellular apoptosis, allowing the bacteria increased time to proliferate within infected cells and/or invade further host cells. The modulation of apoptotic pathways has been observed in other infectious diseases. Chlamydiae prevent the induction of apoptosis through modulation of ERK signalling pathway enabling the bacteria to complete their obligate intracellular growth cycle [31]. *Helicobacter pylori* can induce the formation of an apoptosis complex during infection via ERK signalling [32]. *B. pseudomallei* may also therefore modulate ERK signalling in order to prolong the survival of the host cell.

Additionally, we demonstrated that MCP-1 was rapidly secreted and TNF-α exhibited a more gradual increase throughout the infection of MH-S cells. These two cytokines are key pro-inflammatory markers of infection and have been reported to be upregulated during Burkholderia infection [33]. The activation of ERK is required for the transport of TNF-α mRNA from the nucleus into the cytoplasm and activation of p38, via its downstream substrate MK-2, is linked to increased TNF mRNA stability ([34]) suggesting that the differing phosphorylation states of p38 and ERK seen during *B. pseudomallei* infection are linked to the changes seen in TNF-α secretion.

Finally, our research also aimed to evaluate the importance of ERK in coordinating the immune response to *B. pseudomallei* infection through the use of the inhibitor PD0325901. To our knowledge, this is the first report to describe ERK inhibition in the context of infection with *B. pseudomallei*. We found the phosphorylation of both ERK-1 and ERK-2 within infected MH-S cells was significantly decreased in the presence of the inhibitor. However, no change to P-p38 expression was detected, indicating that PD0325901 did not have a more widespread effect on immune signalling. We found that ERK inhibition following PD0325901 administration led to decreased levels of TNF-α secretion in the supernatant of infected MHS cells. However, despite the decreased ERK activation and reduced TNF-α production observed in PD0325901-treated *B. pseudomallei*-infected cells, this did not afford protection from the bacteria since both treatment regimens with the inhibitor resulted in similar intracellular bacterial counts to untreated cells. It is perhaps not surprising that the administration of a single ERK inhibitor did not alter bacterial clearance of *B. pseudomallei* which has numerous mechanisms for virulence and, in addition, the signalling pathways are complex with both positive and negative feedback mechanisms. There may be sufficient redundancy in the immune response to mitigate the effects of PD0325901 treatment (e.g. ERK has been demonstrated to be activated by both MEK-1 and MEK-2 [29]).

In conclusion, it is clear that *B. pseudomallei* infection causes numerous alterations in proteins involved in intracellular inflammatory cascades. In particularly, we identified that the activation states of both ERK and p38 kinases changed over time as a result of *B. pseudomallei* infection and that these in vitro effects were mirrored within an in vivo context. Specifically, inhibiting ERK activation had no significant effect on the outcome within in vitro models of infection for *B. pseudomallei* and therefore in vivo efficacy was not pursued. More effective approaches are likely to require either the targeting of multiple proteins within a TLR pathway cascade (i.e. overcoming potential redundancies) or alternatively through the use of combination therapies that involve the use of specific inhibitors along with traditional treatments (e.g. antibiotics).
